# Delay

**Published:** 1869-02-01

**Authors:** 


### EDITORIAL.

### *Delay.*

We trust our friends will bear with us a little longer, when we hope to bring the publication of The Journal promptly up to its date. Our personal avocations have been so pressing, that we have scarcely found time to inspect the piles of manuscript which are transformed into our printed pages. The close of the college session, we trust, will afford us more time to devote to editorial work. Meanwhile, the character of the original matter furnished by our numerous correspondents, is such as to render us satisfied, that the editorial pen is not absolutely needed to sustain the interest and importance of the contents of The Journal.

